# About time: how time influences and facilitates patient autonomy in the clinical encounter

**DOI:** 10.1007/s40592-018-0089-7

**Published:** 2019-01-08

**Authors:** Alexis Paton

**Affiliations:** SAPPHIRE Group, Department of Health Sciences, University of Leicester, George Davies Building, Leicester, LE1 7RH UK

**Keywords:** Time, Empirical ethics, Oncofertility, Relational autonomy, Sociology and bioethics, Sociological bioethics, Time and patient autonomy

## Abstract

In this article I discuss the little examined relationship between time and patient autonomy. Using the findings from a study on the experience of premenopausal cancer patients making fertility preservation decisions during their treatment, I focus on how the patients in the study understood time, and how this understanding interacted with and influenced their decision-making. I then analyse in more depth the importance of time in patient decision-making, and the relationship of time to concepts of patient autonomy and decision-making in the field of bioethics more generally. Focusing on the relational conception of autonomy, I conclude that time is an integral part of patient autonomy which warrants further research, such that it can be better integrated into concepts of patient autonomy, and the policy and guidelines that they inform and influence.

## Introduction

Time and patient autonomy are not always academic bedfellows. In many discussions of patient autonomy in bioethics, the concept of time is given little consideration. Some writers, such as sociologists Zerubavel (1979) and Adam (1990), would go so far as to say that the social sciences pay insufficient attention to time. Furthermore, social scientists focus on ‘real time’ or ‘physical time’ and tend to ignore the multifaceted nature of time as a social phenomenon (Adam 1990). Zerubavel argues that time and the study of temporality, as he calls it, may be consistently neglected because time is such “an inherent constituent of social life, and therefore, tends to be taken for granted and ignored as a special focus of attention” (1979, p. xi). By extension, I would argue the same of medical sociology and bioethics; much of the work on autonomy, decision-making and time more generally has focused on time pressures and decision-making, often taking place in the field of psychology, for example, in Decision Field Theory (Busemeyer and Townsend 1993). Zerubavel, who famously studied the role of time in social organisations through an ethnography at a hospital, argues that while the study took place *in* a hospital, his research was not intended to be a study *of* the hospital or medicine with respect to time (1979, p. xvii). In bioethics there is a similar pattern; very little work has focused on the relationship between time and decision-making at all (Scully et al. 2007).

Social time, meaning the way that people interact with the notion of time outside of physical timings or ‘clock-time’, has been a steadily ignored area of study in empirical bioethics. Time is both an integral part of the social and social life. In this view, the way that social time interacts with patient decision-making is an indispensable part of upholding and supporting the autonomous, informed, consent-giving patient. As Adam argues:

Many more aspects of time […] form an integral part of our lives. Some have to do with synchronisation, ordering, sequencing or timing, others with control or measurement, and still others with the time aspects of machines and artefacts. All have a bearing on our lives not as separate abstracted entities but as an interconnected whole. If we accept social science to be about studying, understanding and explaining that reality then we can expect social scientists to take account of time in this multiple and connected way, to know and acknowledge the many aspects of time in their relation and not on an either/or basis (Adam 1990, p. 1).

Time permeates all aspects of human life, and just as Adam has identified its importance to the social sciences, time was an influential factor in how participants of this study made decisions through their diagnosis, treatment and aftercare. Patient experiences reflected Adam’s assessment that ‘we have a relationship to our past, present and future […] Collectively these aspects of time affect the way we see ourselves, our families, our society and our fellow human beings. They influence the timing of our interactions, the way we relate to others, and how we interpret daily and extraordinary events’ (1995, p. 23).

Using research on how women with cancer make fertility preservation decisions prior to cancer treatment (what I will call oncofertility decisions), this article will show how patients have this same active relationship with time. They manipulate it as a way to assert their autonomy, by slowing down something they do not understand, or positioning decisions in a future time when they feel ready to tackle them. In particular I will focus on how the patients in the study understood time and how this understanding interacted with and influenced their decision-making. I will then present a more in depth analysis of the importance of social time to patient decision-making, and the significance of social time to conceptions of patient autonomy and decision-making in the field of bioethics more generally. To begin I outline the background of this work, laying out the framework of the research with regards to patient autonomy, and sociological bioethics, before turning to the research itself.

### A brief glance at patient autonomy

There have been, and continue to be, different understandings of autonomy in bioethics. Perhaps the most well-known and influential of these conceptions, developed by Beauchamp and Childress (2009), is sometimes called the traditional conception of autonomy (Paton 2017, 2018). Advocating that autonomy ought to be respected, such accounts fuse ‘the Kantian concept of respect for persons with Mill’s quite different notion of liberty; that is, persons’ choice of action should not be obstructed unless those actions infringe upon the liberty of others’ (Jonsen 1998, p. 335). Thus, having been operationalized in the informed consent processes, the traditional concept of autonomy dominates patient decision-making in the UK.

Concepts such as relational autonomy, developed by Mackenzie and Stoljar (2000), have presented an alternative understanding that is ‘premised on the shared conviction […] that persons are socially embedded and that agents’ identities are formed within the context of social relationships and shaped by a complex of intersecting social determinants, such as race, class, gender and ethnicity’ (Mackenzie and Stoljar 2000, p. 4). According to the concept of relational autonomy, the ‘self’, at least in part—and, therefore, its autonomy—is derived from relations with others and social institutions. Thus theoretical understandings of autonomy should take these relations into account. When contrasted with traditional theories of autonomy, relational autonomy can be considered to offer a less individualistic and proceduralist account. In this article I will take a relational account of autonomy as the theoretical starting point that best captures how patients act; how they enact their autonomy and make decisions. However, it is unclear whether relational autonomy takes into account the interactions between time and patient autonomy, and so it may not offer a full picture of patient autonomy; a point to which I will return.

### A sociological bioethics

The research presented here also serves as an example of how bioethical inquiry can be ‘done’ under the auspices of sociology. One important aspect of this work is that it takes sociological bioethics (Paton 2018) as its starting point. It examines the bioethical concepts of patient autonomy and patient decision-making through a sociological lens that not only includes sociological methods and methodologies (in this case empirical, qualitative interviews), but also includes social and sociological theory. In this way, the article and the research it reports, aligns itself with the empirical bioethics and sociological bioethics movement that was arguably inaugurated by Erica Haimes (2002) and has since undergone significant development. I have previously argued for the importance of viewing sociology as a constitutive discipline of bioethics in its own right (Paton 2017, 2018), and this article can be considered as an extension of this perspective, serving as yet another example of how bioethics can be ‘done’ using sociology.

Taking a sociological bioethics approach also aligns the methodology of this work with both the relational autonomy framework and Adam’s conception of time. This enables the style of research necessary to access and explore those aspects of the social that Mackenzie and Stoljar (2000) argue are essential components of autonomy, and that Adam argues are necessary aspects of time; namely social relationships, embedded practices and identities, and social determinants such as race and gender.

## Methods and methodology

Using a sociological bioethics approach, the project examined female cancer patients’ experiences of making oncofertility decisions. A qualitative, one-to-one interview based study was designed, such that the lived experiences of those who participated in the study could be prioritised as the primary source of data (Brewer 2000; Mason 2002; Silverman 2004; Paton 2017, 2018). The interviews were structured so as to focus on the social, clinical and ethical concerns of the patients.

After receiving ethical approval from Newcastle University and its associated NHS Trust, participants aged 18–55 were recruited to take part via cancer support groups in Britain. Participants were recruited via support groups for those with cancer. Such groups were identified using websites such as the MacMillan Cancer Support website,^1^ which identifies cancer support groups in specific areas, and via advertisements by local groups who placed notices in hospitals and GP practices. Initially only groups in the Northeast of England were contacted, however due to increasing interest that area was widened to include the Southeast of England as well. These groups were contacted by me, and those who were interested allowed me to make visits to the support group meetings over a series of weeks and months.

When invited to attend a support group I gave short presentations about my research, and interested attendees were given information sheets, a consent form and my contact information. Once written consent had been provided either a phone or face-to-face interview was arranged. No specific type of cancer, cancer stage, prognosis, or remission period was targeted. To meet the inclusion criteria the women had to be premenopausal when they were diagnosed, and had been, or felt they should have been, offered fertility preservation prior to cancer treatment. Eleven patient participants were recruited and interviewed. All interviews were audio recorded with permission, transcribed and anonymised.

Alongside thematic analysis, a grounded theory approach to the analysis of the data was taken. This allowed important issues and major themes present in the data to emerge, which were then organised into major thematic categories (Ghezeljeh and Emami 2009; Attride-Stirling 2001). One of the major themes that emerged from the data, and the focus of this article, was the importance of time in making decisions.

## Time and decision-making

All of the interviewees discussed (unprompted) their experience of time and timing when making medical decisions, marking out “time” as a point of interest and concern for them. Time seemed to permeate all aspects of the patient experience, from waiting to be diagnosed, to worrying about survival, to thinking ahead to a time they may or may not have, to feeling like they did not have enough time to understand, and finally, wondering how to plan their future given all this uncertainty. Time was a vital part of how they made decisions. I would like to focus on how participants interacted with time by discussing three major themes that emerged from the interviews: “Manipulating Time”, “Planning for the Future”, and “Predictive Fuzziness”.

### Manipulating time

In relaying their experiences, interviewees appeared to do something that I describe as “manipulating time”, which they used to justify the decisions that they made. This manipulation came in a number of different forms. For example, some interviewees placed important decisions into a far-off future, not to be dealt with in the present, thus distancing themselves from the decision no matter how urgent the decision may have been. Others reorganised the chronology of events in their retelling so that the story suited and justified the choices that they made. The manipulation of time was often unintentional, occurring in the retelling of their experiences, but served an important function in evidencing their justification for the decisions made. Two interviewees, Stephanie and Heather, participated in this manipulation in contrasting ways.^2^

Stephanie was made aware of fertility preservation, however she decided not to do anything to preserve her fertility. In justifying her decision not to preserve her fertility she describes feeling too young to think about having children, and described putting off those decisions:

We’ll come to that when it comes to that […] I don’t think until the time that I am wanting to have children will it really bother us that much. […] Obviously it will at the time if I do find out that I cannot. But I guess I don’t have to think about it at the minute, I’m too young.

Stephanie’s use of the phrase ‘when it comes to that’ is indicative of one style of time manipulation where interviewees put important decisions into a distant time that had yet to come, and so did not need to be dealt with at present. By putting her difficult decisions about having children into an unidentified future time, Stephanie manipulated time to justify her decision not to make efforts to preserve her fertility during her treatment.

Stephanie did express a desire to have children, but her concerns about whether she will have children seemed to exist in this undetermined future time when she was ‘wanting to have children’. She appeared to be placing that decision in a future time when she would be more able to cope with the prospect of having children. Even contemplating the option of future children (and undergoing fertility preservation) may be to assume too much about a successful treatment. For Stephanie, as long as she never ‘comes to that’, meaning that she is ready to have children, then she never has to consider the consequences of her decision not to preserve her fertility. Nor does she need to consider what decisions that she will have to make about having children in the future. Stephanie’s focus was on getting through her treatment and as a result she remained firmly rooted in the present when she discussed her experience of making oncofertility decisions.

Stephanie also placed her emotions about having children into a future time so as to not deal with them in the present. For Stephanie ‘not until the time that I am wanting children’ would her decision to not preserve fertility really ‘bother’ her. In placing these decisions in a future time, she also distanced herself from her emotions about the decisions that she had already made about not pursuing fertility preservation, a decision that may impact whether or not she will have a choice if and when she does attempt to have children. She manipulated the timing of her decisions to make space for the decisions she will have to make, but also used this manipulation as a coping and even a protecting mechanism against the difficult decisions that she had already had to face and will face.

Another interviewee who manipulated time was Heather. Heather ‘moved’ time around to justify her decisions that went against doctor recommendations. As Heather was pregnant at the time of her diagnosis she had to make a decision about termination. In her interview she described how she manipulated time in making her decision to have her baby before going ahead with her cancer treatment:

I thought well, having the baby is only bagging the time that I would have normally spent if I had had the normal smear test 18 months down the line. Add the 9 month pregnancy, add the 3 month. And I think at the time that’s what I was thinking […] I could have ignored [the smear test appointment]. I could have rang them up, said “Look on your system; I’m not due another 18 months”. “Right, fine.” they could have said to me, and given the time I would have had my baby. And that’s really daft, but that’s how I was thinking.

Heather manipulated time so as to consciously exclude the urgency of her cancer diagnosis and treatment. Heather only found out about her cervical cancer because she had a cervical smear done earlier than scheduled. In her mind the pregnancy would not have been in danger had she not done the smear early, so she decided to carry on as if she had never had the test. The time elapsed was equal. However choosing to ignore her early smear and pretend that she would have one in 9 months’ time, as originally scheduled, resulted in having the time to bring her baby to term, then deal with the cancer. Again Heather manipulated the relationship between decision time and chronological time, but in her case she ‘bagged’ the time she should have had and used it to bring her pregnancy to term.

### Planning for the future

Planning for the future was another way time featured in decision-making for interviewees. Interviewees placed importance on their ability to plan for the future, and linked this planning with the decisions that they would have to make in the future, or to preserve a particular future. Interviewees expressed frustration that planning for the future was often difficult due to the tentative survival percentages given by their doctors, as well as the difficulty in predicting whether they would retain their fertility post-treatment. The emotions expressed by interviewees about their in/ability to plan their futures, and the impact that planning (or the inability to plan) had on their decision-making, indicates its value in helping them make treatment decisions.

For example Robyn felt that planning for her future with her children helped her to make decisions to move forward with her treatment:

And I wasn’t going to not be there for my kids. I was determined that I was going to be there. […] And every time I thought about the future, thought about the kids it was quite difficult because that was the only time I felt any vulnerability. And [the treatment] wasn’t for me, it was for them, because I couldn’t not be here for them.

Robyn had both negative and positive associations with planning for the future that impacted on her decision-making in different ways. Robyn felt she needed to plan for the future as she wanted to be there for her children, making her determined to survive her cancer. In this case, planning for the future, specifically her future with her children, influenced the choices she made about treatment, survival and not pursuing fertility preservation. On the other hand, Robyn could not know for sure that she would survive her cancer. So while she used planning for the future to help her make decisions to move forward with her treatment, she also found that this made her vulnerable. Imagining the future was quite difficult as it required engaging with this vulnerability. Robyn is a good example of how complex the connection is between planning for the future and decision-making. Planning for the future was an integral part of moving forward for many of the interviewees, who, like Robyn, focused on how attaining a future goal affected and influenced their present action and the resulting decisions they made.

Anne also felt that planning for the future was integral to making decisions, but in this case she was thinking about future treatment, and the side-effects of treatment. Anne described how damaging not knowing the future implications of treatments could be for making decisions in the present:

I know like one of our support members […] has had radiation damage [to her reproductive system] […] none of this is kind of explained when they go for it. It’s the end goal that you’re not going to have cancer but some things that you’re left with you know are quite hard to deal with.

Kathleen felt similarly, stating simply about her doctor’s choice to treat her with a radical vulvectomy: ‘My future was never discussed’.

While there is always a level of ontological uncertainty^3^ regarding the side effects of many treatments, Anne felt that not having these “issues” explained leaves the patient in a vulnerable position: they cannot plan for the future, as they are not aware of what the consequences of their decisions will be, and thus struggle to make decisions. Kathleen felt she had even less of an opportunity to discuss her future options than Anne, as her healthcare professionals never discussed with her what life would be like after her radical vulvectomy; simply that it needed to be done to save her life. Many interviewees wanted further discussion with their healthcare professionals about their future (survival, side-effects etc.) than they had received as standard practice, and they viewed their relationship with their healthcare professional as an integral part of accessing this information. This lack of information meant that participants felt oncofertility decisions could not be adequately situated in an appropriate timescale, or within their own personal frameworks, as they had neither the ability, nor the time, to understand the limited information they did receive. Unable to know their survival, their own understanding of their personal relationships, role within their family, job and community, and general understanding of how they as individuals relate to the world was thrown into uncertainty.

Interviewees were also concerned with looking to the future as a way of planning to keep their options open, thus securing the possibility of future decisions after cancer treatment. Angela expressed this best when asked why she would have liked to have known about fertility preservation:

If someone could’ve said “we’re going to take something and keep it to one side” […] Definitely, yeah. […] I would have said yes, keep the bit and then I can make my decisions later.

Planning for the future is similar to the manipulation of time that some interviewees performed, in that patients are making decisions in the present that ensure options to choose from in the future. As Angela explained, fertility preservation would have allowed her to ‘make my decisions later’, by preserving options and choices in a future time when she was ready to make decisions.

Monica also viewed fertility preservation as a way of preserving choice in the future. Monica knew she might want to have children, but at 24 she was not ready to make that decision, and did not want to preserve embryos with the man she was dating at the time she was diagnosed. Despite being pushed to freeze embryos, she pushed back for a better solution that did not force her to make decisions she was not ready to make at that time.

[…] and I hadn’t really given much thought to having children at that point. But I couldn’t say that I definitely didn’t want them […] So when they were saying that we could have had embryos frozen and all that there was just no way that I wanted to do that […] So I just thought well this here Zoladex […] its only short term so I just decided to go with that.

Monica also felt quite strongly that patients should be made aware of all future fertility preservation possibilities so that they can make what she called an ‘informed decision’ about the kind of future they may want and the options that they would like to keep open as a result:

I think so that people can make an informed decision. Rather than, if they’re not made aware of [fertility preservation] and then six months down the line is too late to do anything about it […].

Oncofertility patients are making difficult decisions whereby two time periods need to be taken into account at once: They have to look to the future within the context of their cancer diagnosis *and* their fertility needs, which are not always in sync or fully known.^4^ Interviewees felt that being able to plan for the future was connected to how much information patients receive (and understand) about their diagnosis and treatment. This information could then be incorporated into their individual understandings of themselves, their values and their desires (i.e. whether they were mothers, working, etc.). Alongside the importance of planning for the future, interviewees expressed a need to have as much information as possible about their possible futures. Treatment outcomes were a particular focus, with interviewees wanting to know more about the different outcomes of cancer treatment on fertility, before making decisions that affect the future, and by extension, their fertility in the future. To manage uncertainty individuals will try to reduce the uncertainty of the uncertain thing (Mattias 2010), which may account for the value interviewees placed on information about fertility preservation and cancer when planning for the future.

### Predictive fuzziness

One aspect of planning for the future that may be specific to the medical context is something that Scully et al. (2007) call ‘predictive fuzziness’, a term developed from their work on genetic testing. In that study the term was used to describe how genetic testing cannot tell a patient everything about their disease, its course and severity, for example (Scully et al. 2007).

Likewise, in this study, interviewees experienced predictive fuzziness in several ways. For Mary, the predictive fuzziness about her prognosis was frustrating as she did not know how long she would live, and her doctors were unable to give her an exact timeline, which made it difficult for her to make decisions about keeping or removing her ovaries:

And that’s what I kind of went away and tried to find out about […] everyone wants to know something different and the people are given lots of facts and figures and say “[…] I don’t want to know that I’ve got an 80% chance of living for ten years.” But from my point of view that would have been exactly what I wanted to know and I couldn’t make them tell me, you know I sort of got “Well 50/50. With your ovaries gone you’ve got good things going on”. You know they were very bad at sort of responding to the hard facts.

Here the predictive fuzziness that Mary experienced was due to the conflicting messages that she received from her doctor. On the one hand she had a 50% chance of surviving her cancer, but on the other hand she had ‘good things going on’ if she removed her ovaries. The phrasing of ‘good things going on’ is Mary’s own interpretation of her discussion, and her relationship, with her doctor. However the opaqueness of the statement shows how she interpreted the uncertainty surrounding her survival, which made it difficult to decide if removing her ovaries was worth (to her) this ambiguous ‘good things going on’ promoted by her doctor.

Kathleen’s experience shows how predictive fuzziness can frustrate or even impede decision-making, as she was so unsure if her unborn baby would survive the surgery to treat her cancer that she was unable to plan or make decisions for the birth. Kathleen often mentioned that she was young and uneducated, and so put a lot of faith into her doctor’s authority. When confronted with the predictive fuzziness from him Kathleen’s agency was paralysed, affecting her ability to make any decisions regarding herself or her future child. Her example is, perhaps, extreme. However her reaction to the uncertainty brought on by the lack of information and understanding she had about her disease points to how debilitating predictive fuzziness can be:

I just felt that I couldn’t even go out and buy clothes for this baby, and prepare for him coming […] I just couldn’t. […] probably frightened in case something was drastically going to go wrong. That baby was born and I didn’t even have any new vests […] just didn’t want to know until I knew this baby was alright. And even when I knew the baby was alright, I couldn’t bond, properly.

As Kathleen was the first case of vulval cancer at her hospital, her doctors were unable to give her any indication of her own survival, or that of her baby’s. Kathleen’s uncertainty stemmed from the predictive fuzziness on survival she received from her doctor, which made it difficult for her to make decisions or to act to prepare for her unborn child. As she put it she ‘just couldn’t’.

Predictive fuzziness may be difficult to escape as ‘uncertainty is central to the illness experience’ (Babrow and Kline 2000, p. 1812). Predictive fuzziness is a lack of information that cannot be remedied: it is impossible to give assurance on treatment, success, prognosis or the return of fertility after treatment. For oncofertility patients an aspect of the illness experience is survival, which is very difficult to predict. One participant Brenda had a more matter of fact way of explaining predictive fuzziness, stating: ‘they can’t ever tell any cancer patient, you know, 100%. They’ve always got to say it’s a 99%’.

What is striking about all the interviewees is that the 1%, to use Brenda’s phrasing, often loomed larger in the interviewees’ minds than the 99%. Most interviewees wanted both certainty and clarity about their survival, something that their doctors were unable to give them.

Only one interviewee took a different approach to the predictive fuzziness of her diagnosis and that was Heather. She used the predictive fuzziness to her advantage to make and justify the decision about taking her baby to term before being treated for her cervical cancer. Heather’s wilful, creative interpretation of time allowed her to use the uncertainty of her situation as a means to a desired end. As her doctors could not be sure that her biopsy results were not influenced by her pregnancy hormones, she decided to use that uncertainty as a positive reason to keep her baby:

And he was explaining things […] “you know these biopsies could come back and they could be less […] And the other thing to remember is that when you are pregnant, your hormones, we’re not really getting an exact measurement of what is going on inside there, I want you to keep that in mind”. And I always kept that in mind. And when I did get the [biopsy] results back, and they did say the worst is the worst and it’s what we feared, I always kept that in the back of my head. I’m pregnant, and the hormones […] it can change after 3 months. So I had a lot of things that I think just pacified, or I tried to pacify myself.

Heather used this predictive fuzziness to her advantage. Instead of seeing herself with cancer of an unknown severity, Heather took this to mean that she might not have cancer, or that it might not be severe. She was waiting to be proved wrong in her assessment that ‘it can change after 3 months’ due to her hormones and pregnancy.

Heather was in fact an exception in other ways among the interviewees, in that throughout her diagnosis and treatment she preferred to have the minimum information possible about her situation. She felt that she was unable to cope with knowing all the details and it seemed that she was using this self-imposed lack of information as a protective measure: it was a decision to suspend decision-making until a later time when she had achieved her goal of bringing her baby to term. This is, perhaps, why the predictive fuzziness she encountered did not frustrate her, but instead helped her to justify her actions, and as she said, ‘pacify myself’.

## Discussion

Time was an important part of the interviewees’ experience, and the relationship between decision-making and time/timing was multifaceted. For interviewees the importance of time was not about having the time to make a decision, but about how social time interacted with their perceived futures and the retrospective narratives of their cancer story: all of which patients considered necessary for the decisions that they made. Together all these different ways of interacting and experiencing time amounted to an attempt by the interviewees to cope with the huge amount of uncertainty that cancer diagnosis can bring, and the effect that uncertainty has on patients trying to exercise their autonomy by making difficult, but essential, decisions.

Interviewees often manipulated their understanding of time and timing when reflecting on how and why they made their decisions. Manipulating time not only helped interviewees to explain the actions that they took, but it also helped them feel in control, allowing interviewees to claim some ownership over their actions during a difficult period, and to create a space for future decisions to exist. This space was achieved by breaking up their decisions through time, so that the interviewees gained some control and justification over their decisions, making difficult decisions more manageable and thus possible to make. The creation of this space also gave participants agency over the decisions they had made and the decisions they may have to make.

This argument is supported by Scully et al.’s (2007) suggestion that the manipulation of time allows patients ‘moral space’ within which to make their own difficult decisions and exercise their agency. Scully et al. (2007) found similar time manipulation in their study of patients undergoing genetic testing. They describe a scenario whereby their participants manipulated the relationship between decision time and chronological time to create moral space by ‘an active manipulation of their subjective experiences of time passing, by fractioning the anticipated future into an immediate step and further steps that could be thought about later.’ (Scully et al. 2007, p. 211).

By adopting a cross-that-bridge-when-I-get-there approach patients are shifting their temporal field such that it ‘separates difficult moral decisions that in practice are interlinked’ (Scully et al. 2007, p. 214). This type of ‘fractioning’, as described by Scully et al., is similar to the way that interviewees in this study assigned their decisions to a future unknown time. The nature of fertility helps to facilitate this cross-that-bridge approach adopted by the participants, as the loss of fertility is difficult to predict, and most individuals can only be diagnosed as infertile once they have started trying for a pregnancy: i.e. when they become ready to cross the bridge.

Manipulating time served to facilitate, justify, cope with, and protect the interviewees from decisions that had to be made, allowing them to feel as if they were still maintaining some control. In doing this, interviewees were creating the ‘moral space’ necessary to exercise their autonomy and make difficult decisions themselves.

Interviewees needed that space to deal with the difficulty they had in planning for the future. They often had to make decisions about things that had yet to happen or that they had not even thought about yet (for example, whether they wanted to have children). Planning for the future is particularly hard for oncology patients as their prognosis is uncertain, making it difficult for them to make decisions about their future when they cannot know if they will survive to that future. They are simultaneously experiencing a lack of information and an uncertainty of survival. These decisions were doubly difficult when considering fertility preservation as patients have to make decisions about their future survival, as well as their future fertility, and these two futures are not always compatible.

An integral part of moving forward for interviewees was planning for the future, and in making decisions they focused on how the future affects and influences their present action. Planning for the future had a number of positive consequences for interviewees that helped facilitate their autonomy: for example it could be about hope, about attending an event in the future, or be as simple as choosing a cancer treatment based on the future side-effects. Planning for the future was also a way for interviewees to preserve and postpone options and choices for themselves in the future that they were not prepared to make a decision about in the present.

Planning for the future is also how patients ‘colonize’ that future, whereby individuals extend or project their consideration of present needs to include consideration of their future needs (Hagerstrand 1985; Adam 1990). Patients do this for the same reason that all people do, to try and secure the safety of their futures and eliminate uncertainty (Adam 1990). For oncofertility patients, fertility preservation is a way of ensuring one’s imaginary future existence by such colonisation, thus securing both future fertility and future options and choices about fertility.

Similarly, while planning for the future was experienced by participants as the enacting of patients’ autonomy based on the information they are given and understood, predictive fuzziness was experienced by the patient as a lack of information and understanding about their future that had the potential to thwart patient autonomy. Uncertainty due to predictive fuzziness about their prognosis was a key feature of interviewees’ experience of time in the decision-making context, and how they constructed, or could not construct, the long term framework within which all people make decisions (Scully et al. 2007). ‘Knowing’ their futures was understood by the interviewees as having information on their current condition that they could use to make decisions moving forward through time in a way that made them feel comfortable and in control of their lives, an affirmation of the agency that they felt was slipping away. Predictive fuzziness meant that their futures were sometimes impossible to know, as their prognosis was unknown, effectively eliminating a source of information that patients consider important when making decisions.

Uncertainty is often related to ‘the sufficiency, reliability and validity of information’ (Babrow and Kline 2000, p. 1810), and predictive fuzziness in relation to a cancer diagnosis means uncertainty about survival, thus making decisions about the future difficult. Patients value predictive information about their disease when planning, preparing and making decisions about the future. For participants predictive fuzziness became a form of epistemological uncertainty; uncertainty about specific information and the applicability of that information (Mattias 2010). Interviewees experienced this epistemological uncertainty alongside the existing ontological uncertainty that everyone experiences as part of living in the world and the impossibility of knowing everything (Mattias 2010). Interviewees expressed a frustration with epistemological uncertainty as they felt that they did not have sufficient information (as patients) about survival and treatment, making it difficult, if not impossible for them to make decisions and, by extension, to exercise their autonomy.

This uncertainty influences patient decision-making as it acts as a road block to achieving the informed understanding that patients feel is critical to decision-making, and which is also considered a crucial criterion of most conceptions of patient autonomy (Mackenzie and Stoljar 2000; Beauchamp and Childress 2009; Paton 2017). In oncofertility the combined predictive fuzziness of cancer prognosis and future fertility is impossible to rectify as there is *no* way of knowing with certainty that a patient will survive treatment *with* their fertility intact. Interviewees felt that not understanding, or not knowing all the information about the various available futures (in terms of fertility preservation, treatment outcomes and treatment side-effects), made it difficult to know which future to aim for and thus how to tailor their actions in the present. Given the importance of informed understanding to the concept of patient autonomy as a whole, the ramification of medical uncertainty like predictive fuzziness requires further research to better understand how uncertainty and time interact with patient autonomy.

While this may seem a small point about information access, it is actually part of a larger issue concerning the facilitation of patient autonomy with regards to time. As Scully et al. (2007) found, informed consent is built on the principle that patients understand the information given to them, and that they continue to understand the information and have access to this information throughout the decision-making process: i.e. understanding is developed over time, it persists through time and in future time. However previous research has found that, in practice, patients have difficulty accessing and understanding medical information, and that while emphasis has been put on allowing more time for continuous access to information, patients vary in their ability to gain access to, and understand, information (Towle et al. 2006; Martinez et al. 2009; McMullen 2012; Zikmund-Fisher et al. 2012; Kim et al. 2013).

Additionally, since much of cancer treatment and fertility preservation occurs over a period of time, it is necessary to extend the level of choice across this period of time as well; to take what Scully et al. (2007) argue is the ‘longer perspective’. Decisions in cancer treatment are simultaneously decisions for the now and for the future. These patients exist in two temporal arenas: their ‘now’ selves and their ‘future’ selves. They balance and compromise between the two, as cancer treatment decisions affect the availability of future fertility options. The decisions that oncofertility patients make in the ‘now’ are influenced by past decisions, and help them to ‘colonize the future’ (Hagerstrand 1985), projecting their decisions forward in such a way that they feel comfortable with the choices they have made. These three temporal arenas of past, present and future make up the long-term framework within which patients make decisions. Taking the longer perspective also respects the various relational influences on autonomy that are constantly at play, and ever changing, for the cancer patient. This includes things like social conventions about having children, expectations of the cancer patient’s role in their families and in society, and the dynamic power relationship between the patient and the oncologist. All of which will help to make up the patient’s long-term framework, which they use to make decisions.

Adam describes this process of travelling across the long-term framework as ‘the power of the human mind to visit past events, to re-invent them, create alternative versions and plan a multitude of futures. We are able to imagine the world in a projected future-present upon which we can reflect and make out choices’ (Adam 1995: 18). Despite its importance, the medical context often truncates this framework, requiring decisions that affect the future to be made acutely, with little time for the necessary reflection. As a result the decisional framework suddenly becomes very small and very immediate, effectively excluding the long-term framework altogether.

While many bioethical concepts of autonomy consider more than the immediate space around these acute moments of decision-making, most focus on the legitimacy of the acute decision being made when outlining their criteria for/description of autonomy in medicine (consider, for example, the emphasis placed on competence and informed consent at the *exact* moment of consent). Doing this focuses on what is needed for the patient to maintain autonomy at the point of decision-making, but not on how the patient can maintain their autonomy within the context of the patient’s long-term framework. This is a point supported by Adam who argues that individuals ‘create their futures as a continuing affair in the present […] the future is no longer merely predicted, it is actively constructed’ (Adam 1990, p. 98). Medical decisions are not discrete points in time that can and should be separated out from each other. Instead, building on Adam’s argument, decisions made within the medical context are like any other decision made in an individual’s life, and are thus part of and influenced by the ‘continuing affair’ that is that person’s life, an affair whose importance is recognised in relational accounts of autonomy.

Adam’s phrasing of ‘actively constructed’ implies that agency is embedded within the long-term framework, and decisions that are made within its scope are a reflection of that agency. Taking account of a patient’s long-term framework is an affirmation of their agency over their own lives, and allowing them to colonize their medical future (to borrow and bend Hagerstrand’s phrase) is a facilitation of their autonomy in the clinical encounter.

Other aspects of a person’s long-term framework are arguably their relationships, their social context, culture, gender and identity. These are all aspects of a person’s life that influence their decision-making, and this belief is the core of relational autonomy (Mackenzie and Stoljar 2000). Time is not as prevalent in relational accounts of autonomy, but that is not to say that the importance of time cannot be easily incorporated into relational autonomy. As Benner and Wrubel (1989) argue in their account of caring, time is crucial as it is part of our very being. It is a phenomenon as present in our lives as those listed by Mackenzie and Stoljar. Hall articulates this best, arguing that ‘[t]ime is relational, circular and creates meaning, and it combines past, present and future’ (Hall 2005, p. 95). (Social) time is more than clock-time, it is a crucial link between what Hall calls ‘being and doing’. In this sense time is an integral, essential force in autonomy, reflecting autonomy’s relationality, its complex relationship between agency and action, and the operationalisation of that autonomy across the long-term framework within which every person makes decisions. Time and autonomy cannot, and should not, be separated out from each other, and theories of autonomy should incorporate time into their accounts to reflect this essential relationship between the two.

## Conclusion

The continued refrain throughout this research was that oncofertility decisions, and the resulting fertility preservation, was seen as choice preservation for the future. Fertility preservation allowed patients to plan for the future without having to commit to a child, just to the (future) option of having a child. The focus of interviewees was less on the ability to have children after cancer but, instead, on the ability to choose to have children after cancer, if that was what they decided: a subtle but important distinction that would not force patients to make decisions that they were not ready to make.

When interviewees felt uncomfortable making decisions they attempted to manipulate time to gain the moral space necessary to make their decisions. Interviewees felt that patients need the time to plan for the future in order to make decisions in the present. This is part of their temporal understanding of the long-term framework within which they make decisions. To make these decisions, patients want and need information about their futures. This information is not always easily available due to the predictive fuzziness that cancer diagnosis imposes on the patient.

Patients also use time to assert their autonomy by creating space in which to make decisions, or ‘keep’ decisions until a later time. Interviewees made it clear that having more time would have helped them to make decisions with which they were comfortable. I interpret this as their own attempts at supporting and respecting their autonomy. Most interviewees felt that a better relationship with their doctors would facilitate this, as a good relationship with their doctor would facilitate a continued understanding of information throughout the medical encounter, thereby creating more ‘moral space’ for the patient to exercise autonomous decision-making.

This continuous understanding of, and interacting with, information over time is a vital part of maintaining and facilitating patient autonomy. However many theoretical conceptions of autonomy focus only on the point of decision-making, and not on the moments that precede and proceed from it. Failure to facilitate patients’ continuous informed understanding is a failure to acknowledge the long-term frameworks in which patients make decisions. Clinicians and ethicists often see the decisions to be made as close in time to the clinical encounter, but to the patient the whole process has been going on longer than the point of contact with the clinician, and will continue on after as well. It is important to allow for the patient’s long-term perspective as it may be necessary for the patient to repeatedly review the information so that they feel they understand it within their long-term framework.

Respecting the long-term framework in which the patient makes decisions is important to consider not only when respecting patient autonomy, but also when facilitating patient autonomy. It is not just the clinical encounter, but the patient’s whole life story, values, and beliefs for their present and future that inform their decisions. These values and beliefs are aspects of decisions that will be made about fertility preservation and cancer treatment that exist totally outside the clinical encounter, but reside comfortably in the patient’s long-term framework, contributing to how she asserts her agency, moral competency and autonomy.

Social time and decision-making are interconnected in ways that are integral to patient autonomy. How an individual understands and interacts with time is important for understanding and exercising their autonomy, as their understanding of time ‘constitutes the difference between having choices and seeing one’s social life as determined.’ (Adam 1990, p. 5). Seen through the lens of bioethics this distinction can be understood as the difference between autonomy (what Adam calls ‘having choices’) and non-autonomy (what Adam calls ‘seeing one’s life as determined’). If autonomy and being autonomous are to continue as fundamental concepts in bioethics, then the relationship between autonomy and social time should also be valued, and by extension social time should be a substantial feature of interest to bioethics research in the future.

## Footnotes

1 https://www.macmillan.org.uk/information-and-support/coping/talking-about-cancer/find-groups-and-talks-near-you [Accessed: Date,—you might want to put first accessed i.e. when you were doing the research, and last accessed i.e. is it still functional.].

2 As these interviews best exemplify the “manipulation” of time I take them as my focus. Nevertheless, the other interviewees described similar instances of manipulating time.

3 By ontological uncertainty I am referring to the uncertainties that exist because uncertainty is a fundamental part of being in the world and it is impossible to know everything. For more on ontological uncertainty see Matthias (2010, p. 201).

4 By this I mean that fertility preservation is incompatible with certain cancers. For example, if the cancer is so advanced and aggressive that chemotherapy cannot wait the 6–8 weeks required for egg retrieval, or if the treatment requires the surgical removal of the uterus, ovaries or cervix, or if the cancer is oestrogen receptor positive (in which case the process of retrieving eggs would actually stimulate the growth of the cancer).
